# The effect of virtual specialist conferences between endocrinologists and general practitioners about type 2 diabetes: study protocol for a pragmatic randomized superiority trial

**DOI:** 10.1186/s13063-022-06961-y

**Published:** 2022-12-28

**Authors:** Thim Prætorius, Anne Sofie Baymler Lundberg, Esben Søndergaard, Søren Tang Knudsen, Annelli Sandbæk

**Affiliations:** 1 Steno Diabetes Center Aarhus, Aarhus University Hospital, Palle Juul-Jensens Boulevard 11, 8200 Aarhus N, Denmark; 2 Research Unit for General Practice, Aarhus University, Aarhus, Denmark; 3 Department of Public Health, Aarhus University, Aarhus, Denmark

**Keywords:** Virtual conferences, General Practitioners: General practice, Randomized trial, Type 2 diabetes

## Abstract

**Background:**

To support the primary care sector in delivering high-quality type 2 diabetes (T2D), literature reviews emphasize the need for implementing models of collaboration that in a simple and effective way facilitate clinical dialogue between general practitioners (GPs) and endocrinologists. The overall aim of the project is to evaluate if virtual specialist conferences between GPs and endocrinologists about patients living with T2D is clinically effective and improves diabetes competences and organization in general practice in comparison to usual practice.

**Methods:**

A prospective, pragmatic, and superiority RCT with two parallel arms of general practices in the Municipality of Aarhus, Denmark. All general practices are invited (*n* = 100). The intervention runs for 12 months and consists of four virtual conferences between endocrinologists and an individual general practice. Before the first conference, an introductory webinar teaches GPs about how to use an IT-platform to identify and manage T2D patients. The main analysis (month 12) concerns the difference between the intervention and control arm. It is expected that the virtual conferences at the patient level will improve adherence to international recommendations on diabetes medication for T2D patients and improve the risk profile with a reduction in glycated haemoglobin, blood pressure, and cholesterol. The study design allows for identifying a significant difference between the intervention (*n* = 15) and control group (*n* = 15) regarding the three primary clinical outcomes with a power of 0.8870–0.9941. At the general practice level, it is expected that general practitioners and practice staff in the intervention group will improve self-reported diabetes competence and organization. The control arm will get the intervention when the primary intervention ends (months 12–24), and the intervention arm transitions to a maintenance phase.

**Discussion:**

The potential of virtual conferences is yet to be fully tapped because of methodological limitations. Studies have also not yet systematically evaluated virtual conferences in the context of chronic care using a high-quality research design. Given the nature of this real-life intervention, general practitioners and endocrinologists cannot be blinded to their allocation to either the intervention or comparison arm.

**Trial registration:**

ClinicalTrials.gov, United States National Institutes of Health trial ID: NCT05268081. Registered on 4 March 2022.

**Supplementary Information:**

The online version contains supplementary material available at 10.1186/s13063-022-06961-y.

## Introduction

### Background

Globally, 462 million people is estimated to live with type 2 diabetes (T2D). This corresponds to 6.3% of the world’s population, which makes T2D one of the most common chronic diseases [1]. In Denmark, 240,000 people (4.1%) are diagnosed with T2D, and by 2030, the number is projected to nearly double [2]. T2D elevates the risk of developing macro- and micro-vascular complications (e.g., atherosclerotic cardiovascular disease, chronic kidney disease and peripheral neuropathy) resulting in higher mortality, higher morbidity, increased disability, poorer quality of life [3], and a substantial economic burden for society in medical costs and reduced productivity [4]. The risk of developing complications can be considerably reduced if health care professionals help to secure optimal levels of lipids, blood pressure, and glycated haemoglobin by appropriate pharmacological treatment to supplement life-style interventions [3, 5]. However, management of T2D is a complex task since it is often accompanied by other diseases (e.g., heart or kidney failure) [6–8], which requires the use of several different and sometimes interacting drugs. Furthermore clinical guidelines are revised regularly as new knowledge and new pharmaceuticals emerge at a rapid pace [9, 10]. Unfortunately, these care challenges have led to a wide gap between the recommended pharmacological care and the care that patients receive, both internationally and in Denmark [11–13]. Thus, it is urgent to improve the pharmacological management of patients living with T2D.

In Denmark, most patients with T2D are treated and followed by their general practitioner (GP). Hospital-employed endocrinologists provide written support and phone counselling to GPs regarding treatment of patients with T2D. To support GPs in providing high quality care of T2D, literature reviews emphasize the need for implementing models of collaboration that in a simple and effective way facilitate clinical dialogue between GPs and endocrinologists [3, 14]. Previous studies have tried various quality improvement strategies targeting health care providers or the organization of health care delivery, of which the latter is found to have the largest effect on disease management [15, 16]. Presently, virtual conferences are being tested across healthcare systems as a new way connecting hospitals and general practices [17–21] because of its potential to improve treatment, increase competences in general practice, and provide cost-effective health care [22]. This potential, nevertheless, is yet to be fully tapped and studied because of methodological limitations such as lacking a control group or an adequately powered multi-group trial [15]. Moreover, studies have not systematically tested and evaluated virtual conferences in the context of chronic care using a high-quality research design [14] or done it by studying the effect on clinical endpoints and competences in general practice [23]. A prospective study suggests that cross-sectoral virtual conferences about T2D care improve metabolic and hemodynamic parameters after 1 year [24].

The aim of the project is to evaluate if virtual specialist conferences between endocrinologists and general practitioners about patients living with T2D is clinically effective and improves diabetes competences and organization in general practice in comparison to usual practice. This aim will be studied in a pragmatic randomized controlled trial design that maximizes external validity, is tested in a wide range of participants, and measures important clinical outcomes [25]. Since our intervention will influence the general practice as a whole, it will be the unit of randomization. The intervention was developed using the Medical Research Council (MRC) Framework [26, 27]. The project also studies factors that facilitate or limit the implementation of cross-sectoral and virtual specialist conferences [28], which are important to understand for subsequent upscaling [18]. The project will provide new and clinically relevant knowledge on how to manage the increasing number of persons with T2D seen in general practice.

### Study aim and objectives

The overall aim of the project is to evaluate if virtual specialist conferences between endocrinologists and general practitioners about patients living with T2D is clinically effective and improves diabetes competences in general practice in comparison to usual practice. We distinguish between clinical objectives and competence and organization objectives in the following ways.

The primary clinical objective is to study if the intervention group compared to general practices receiving usual practice is superior in improving the percentage of patients with T2D and three types of co-morbidities on appropriate diabetes related medication:

- Ischemic heart disease and/or stroke being treated with glucagon-like peptide 1 receptor agonists (GLP1-RA) and sodium glucose cotransporter 2 (SGLT2) inhibitor
- Micro/macro-albuminuria being treated with Angiotensin-converting-enzyme-inhibitor (ACE) or angiotensin-2-receptorantagonist (AT2)
- Low-density lipoprotein (LDL) > 2.5 mmol/L being treated with Statins.

The secondary clinical objective is to study if the intervention group compared to the control group decreases the percentage of patients with type 2 diabetes and respectively:

- HbA1c < 58 mmol/L
- HbA1c < 53 mmol/L
- Blood pressure < 140 mmHg
- Blood pressure < 130 mmHg
- LDL > 2.5 mmol/L
- LDL > 1.8 mmol/L

The primary diabetes competence and organization objective is to study if general practitioners’ in the intervention group compared to the control group self-reports a higher degree of:

- Confidence and skills in managing type 2 diabetes in general
- Confidence in managing type 2 diabetes and cardiovascular disease or heart failure
- Confidence in managing type 2 diabetes and blood pressure
- Confidence in managing type 2 diabetes and kidney disease
- Confidence in managing type 2 diabetes and cholesterol.

The secondary diabetes competence and organization objective is to study if the intervention group compared to the control group self-reports a higher assessment of:

- General practitioners’ rating of relational coordination in the general practice
- General practitioners’ rating of relational coordination with the hospital
- General practitioners’ rating of using virtual conferences
- Practice staffs’ rating of relational coordination in the general practice
- Practice staffs’ rating of relational coordination with the hospital
- Practice staffs’ rating of using virtual conferences
- Practice staffs’ confidence in managing type 2 diabetes in general
- Practice staffs’ confidence in managing type 2 diabetes and cardiovascular disease or heart failure
- Practice staffs’ confidence in managing type 2 diabetes and blood pressure
- Practice staffs’ confidence in managing type 2 diabetes and kidney disease
- Practice staffs’ confidence in managing type 2 diabetes and cholesterol

### Trial design

**Fig. 1:**
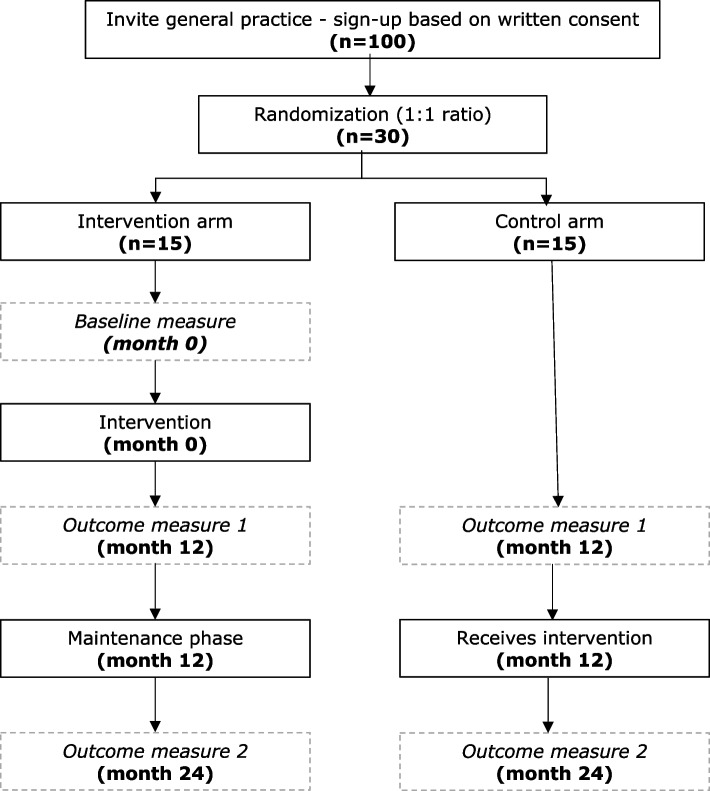
Study flow chart

A prospective, pragmatic, and superiority RCT with two parallel arms of general practices. General practices will be randomized to the virtual conference intervention or usual care arm with 1:1 allocation, stratified by the type of general practice to ensure a balanced allocation. Additional file 1 reports the trial according to the *Standard Protocol Items: recommendations for Interventional trials* (SPIRIT) statement [29]. Figure 1 shows the study flow chart.

## Methods

### Trial setting

Denmark is a decentralized health system where the national government provides block grants from tax revenues to the five regions and 97 municipalities who deliver health care services. All residents are entitled to publicly financed health care. The five regions are responsible for hospital care, including emergency care, psychiatry, and for health care services provided by general practitioners (GPs) and medical specialists working in private practice. Danish GPs (*n* = 3,326) are self-employed, predominantly work in partnership practices, and work on contract for the public funder [30, 31]. Most patients with T2D are followed in general practice. Endocrinologists working in hospitals provide written support and phone counselling to GPs regarding treatment of patients with T2D.

### Trial participants

General practice: All general practices (including general practitioners and clinical staff) located in the Municipality of Aarhus are invited (*n* = 100). Participation is remunerated. Hospital: Endocrinologists from Steno Diabetes Center Aarhus, Aarhus University Hospital.

### Eligibility criteria

General practices are eligible for inclusion if licensed and located in the municipality of Aarhus. GPs must consent to participate in the intervention and collect the data needed to measure patient and general practice outcomes. A hospital secretary will collect the signed informed consent to participate in print before randomization. General practitioners must be willing to bring patient cases to the virtual conferences who are ≥ 18 years, diagnosed with T2D and who they would like to discuss with the endocrinologist.

The endocrinologists from Steno Diabetes Center Aarhus must be trained as endocrinologist with speciality in diabetes and provide written informed consent to participate in the intervention.

### Intervention

#### Method used for the intervention development

The intervention was developed using the Medical Research Framework for developing complex interventions [26, 27]. The intervention development process (Additional file 2) consisted of iterative cycles of adjusting the intervention, in which two approaches were combined to gain methodological strength: a partnership- and evidence-and-theory based approach [32]. The evidence-and-theory based approach consisted of using the behaviour change wheel method including a COM-B (capability, opportunity, motivation, behaviour) in addition to the MRC framework [33]. Throughout the process, a programme theory guided the intervention development.

Small-scale feasibility tests had been conducted in other municipalities from the Central Region Denmark, which the municipality of Aarhus also belongs to. Evaluations of the feasibility tests showed that the intervention was implementable in daily clinical practice and that general practitioners, practice staff, and endocrinologist were satisfied with the content and results of the intervention.

#### Intervention arm

Table 1 provides a detailed description of the intervention according to the *Template for Intervention Description and Replication checklist and guide* (TIDieR) [34]. The intervention consists of four virtual conferences (45 min) between endocrinologists and an individual general practice that take place over a period of 12 months (month 2, 5, 8, and 11). Before having the first virtual conference, an introductory webinar (90 min in month 1) is held to learn GPs about how to use the electronic “Diabetes Overview” to identify and manage patients with T2D.

**Table 1:** Intervention according to TIDieR (Template for Intervention Description and Replication)

Brief name	Virtual specialist conferences between general practitioners and endocrinologists about type 2 diabetes
Why	To support general practitioners (GP) in managing their patients living with type 2 diabetes (T2D). To optimize pharmacological treatment of patients with T2D
What	Virtual conferences between GPs and endocrinologists lasting approx. 45 min. The four conferences are thematic: (1) T2D and cardiovascular disease and heart failure, (2) T2D and cholesterol and lipids, (3) T2D and kidney disease and blood pressure, and (4) T2D and a free topic. The GP is asked to bring to each conference 2–3 patient cases related to the theme and 1–2 patient cases of their own choice. Each conference follows the same format: (1) check in (5 min), (2) presentation by endocrinologist on the conference theme with a focus on medication and treatment guidelines (10 min), (3) presentation by GP and joint dialogue about 2–3 patients related to the theme (20 min), (4) presentation by GP and joint dialogue about 1–2 patients unrelated to the theme (optional; 5 min), and (5) wrapping up and summary of learning points (5 min) Before the first virtual conference, an introductory webinar (90 min) is held to learn GPs about how to use the electronic “Diabetes Overview” to identify and manage patients with T2D All conferences and webinars are carried out according to a manual
Who provided	Preparation and roles before and during virtual conferences: GP(s): - Chair of the meeting - Finds patient cases via “Diabetes Overview” - Presents patient cases General practice staff: - Participates in the dialogue Endocrinologists from Steno Diabetes Center Aarhus. Each general practice meets the same endocrinologist in all the conferences: - Presentation on the conference theme - Dialogue about and advises on patient cases
How	Virtual conferences between one endocrinologist and at least one GP from each general practice. MDs in training and practice staff from the general practice is encouraged to participate The virtual conference is integrated into the working plan of the endocrinologists GPs are responsible for planning their own clinical programme
Where	Virtual meeting platform. The endocrinologists will be sitting at Aarhus University hospital. The GPs will be sitting in their practice or at home
When and how much?	Each general practice will receive four conferences in 1 year each lasting approx. 45 min and one introductory webinar (90 min)
Tailoring	GPs decide themselves on which patient cases they want to bring to the conferences: 2–3 related to the theme and 1–2 patient cases of their own choice
Modifications	Modifications due to changing circumstances or on behalf of the participants will be noted throughout
How well	Adherence and fidelity in the outcome study is analysed, and the trial will study process outcomes

Each general practice is assigned to an endocrinologist who they meet at all four conferences. The four virtual conferences are thematic: [1] T2D and cardiovascular disease and heart failure, [2] T2D and lipids, [3] T2D and kidney disease and blood pressure, and [4] T2D and a free topic selected by the GP. The GP is asked to bring two–three patient cases to each conference who are related to the theme and one-two patient cases of their own choice. The GP chairs the meeting. Each virtual conference follows the same format: [1] check in (5 min); [2] short presentation by the endocrinologist on the conference theme with a focus on medication and treatment guidelines (10 min); (3) presentation by GP and joint dialogue about 2–3 patients related to the theme (20 min); (4) presentation by GP and joint dialogue about 1–2 patients unrelated to the theme (optional; 5 min); and (5) wrapping up and summary of learning points (5 min).

Conferences and webinars are carried out according to a manual. The intervention will not be modified during the study period. The general practices in the intervention group will transition to a maintenance phase (month 12 to 24) where they get a maximum of two virtual specialist conferences.

#### Comparison arm

General practices allocated to the control group will continue to get access to the usual written or telephone support by an endocrinologist in hospitals. This requirement to provide diabetes support to GPs is part of a collective agreement between the Danish Regions and the Association of General Practitioners [35]. The control group will get the intervention after the primary intervention ends, that is, after month 12.

#### Concomitant hospital support

In both arms, general practices can contact the hospital and the Steno Diabetes Center Aarhus as they please. The frequency of other contacts will be measured in the survey to GPs.

### Assignment of interventions

Block randomization is performed at the general practice level. General practices will be randomized in a 1:1 ratio to either the intervention or the control group by a statistician, according to a computer-generated list, independent of the measurement team. Randomization included stratification by number of full-time GPs and geographic location. The latter because general practices located in the centre and periphery of the municipality of Aarhus differ in patient demographics. A hospital secretary will be in charge of enrolment of participants.

### Outcomes

#### Clinical outcomes

Table 2 shows the primary and secondary patient outcome measures. The primary patient outcomes are the percentage of patients with T2D and three types of co-morbidities on appropriate diabetes-related medication: ischemic heart disease and/or stroke; micro- or macro-albuminuria; and LDL > 2.5 mmol/L. To account for multiplicity, we use the Bonferroni correction method to adjust the *p*-values of the three primary clinical outcomes. The Bonferroni correction sets the significance cut-off at α/n [36], which means we only reject the null hypothesis of a primary clinical outcome if the *p*-value is less than 0.0167 (i.e., 0.05/3).

**Table 2:** Overview of primary and secondary outcomes measures

Aspect	Outcome measure
Primary clinical outcome measures	Percentage of patients with T2D and ischemic heart disease and/or stroke being treated with glucagon-like peptide 1 receptor agonists (GLP1-RA) and sodium glucose cotransporter 2 (SGLT2) inhibitor
	Percentage of patients with T2D and micro/macro-albuminuria being treated with angiotensin-converting-enzyme-inhibitor (ACE) or angiotensin-2-receptorantagonist (AT2)
	Percentage of patients with T2D and LDL > 2.5 mmol/L being treated with statins
Secondary clinical outcome measures	Percentage of patients with T2D and HbA1c < 58 mmol/L
	Percentage of patients with T2D and HbA1c < 53 mmol/L
	Percentage of patients with T2D and blood pressure < 140 mmHg
	Percentage of patients with T2D and blood pressure < 130 mmHg
	Percentage of patients with T2D and low-density lipoprotein > 2.5 mmol/L
	Percentage of patients with T2D and microalbuminuria and LDL > 1.8 mmol/L
Primary competence and organization outcome measures	The extent to which the GP is confident managing T2D in general
	The extent to which the GP is skilled in making decisions on T2D in general
	The extent to which the GP is confident managing T2D and ischemic heart disease, stroke, peripheral artery disease, and heart failure
	The extent to which the GP is confident managing T2D and blood pressure
	The extent to which the GP is confident managing T2D and kidney disease
	The extent to which the GP is confident managing T2D and cholesterol
Secondary competence and organization outcome measures	GPs rating of the degree of relational coordination within the general practice
	GPs rating of the degree of relational coordination with the endocrinology department
	GPs rating of using virtual conferences based on the Technology Acceptance Model
	The extent to which the practice staff is confident managing T2D in general
	The extent to which the practice staff is confident managing T2D and ischemic heart disease, stroke, peripheral artery disease, and heart failure
	The extent to which the practice staff is confident managing T2D and blood pressure
	The extent to which the practice staff is confident managing T2D and kidney disease
	The extent to which the practice staffs is confident managing T2D and cholesterol
	Practice staffs’ rating of the degree of relational coordination within the general practice
	Practice staffs’ rating of the degree of relational coordination with the endocrinology department
	Practice staffs’ rating of using virtual conferences based on the Technology Acceptance Model

The secondary patient outcomes concern the percentage of patients with T2D alongside, respectively: HbA1c < 58 mmol/L, HbA1c < 53 mmol/L, blood pressure < 140 mmHg, blood pressure < 130 mmHg, low-density lipoprotein (LDL) > 2.5 mmol/L, microalbuminuria, and LDL > 1.8 mmol/L.

#### Competence and organization outcomes

Table 2 shows the primary and secondary outcome measures at the general practice level. The primary outcome concerns the competence of the GPs with regards to managing T2D and their patient population living with T2D. The secondary outcome measures are (a) the competence of the practice staff with regards to co-managing patients with T2D and (b) GPs and practice staffs’ rating of the degree of relational coordination within the general practice, their rating of the degree of relational coordination with the endocrinology department, and their rating of using virtual conferences.

### Recruitment strategy for achieving adequate enrolment

The recruitment strategy relies on six elements. First, GPs are reimbursed for participating in the conferences, collecting data and answering the survey. Second, formal collaboration with the chairman of the Local Association of General Practitioners in Aarhus municipality (PLO-Aarhus) and a representative of the Regional Association of General Practitioners from the Central Denmark Region (PLO-M). Third, regional-level support through a project grant from the Regional Committee for Quality and Development in General Practice to reimburse GPs for their participation in the study. Fourth, two types of official PLO newsletters (one from PLO-Aarhus and another from PLO-M) containing information about the project and how to participate is distributed to GPs in Aarhus Municipality. Fifth, GPs will receive project information through ground mail and electronic mail (via the Danish public electronic mailbox system, e-boks Business) using publicly available data from the Central Business Registration. Sixth, GPs will learn about the project by calling them on telephone. The GPs will be contacted in a random order by generating a computerized random sequence of ID numbers from 1 to 100.

### Retention

We will continuously monitor the trial for any operational issues (e.g., failure in appointment management, IT-issues). We will communicate timely and directly with the enrolled general practices. As regards data collection of quantitative and qualitative data, data will be provided by participants who are remunerated, thereby increasing the availability of data. We will reduce participant burden by using an electronic survey and keeping questionnaires as short as possible. To encourage retention at point of data collection, we will send out up to three reminders via e-mail and phone.

### Blinding

Given the nature of this real-life intervention, general practitioners and endocrinologists cannot be blinded to their allocation to either the intervention or comparison arm. To limit influencing the behaviour of the control group, baseline information will not be collected from them and are not informed about the type of data that are collected for the main analysis at month 12. Researchers analysing the data will be blinded regarding which arm each general practice belongs to.

### Data collection

Table 3 shows the study schedule including data collection points according to the SPIRIT guideline. Data are collected at month 0, 12, and 24 for the intervention group and month 12 and 24 for the control group.

**Table 3:**
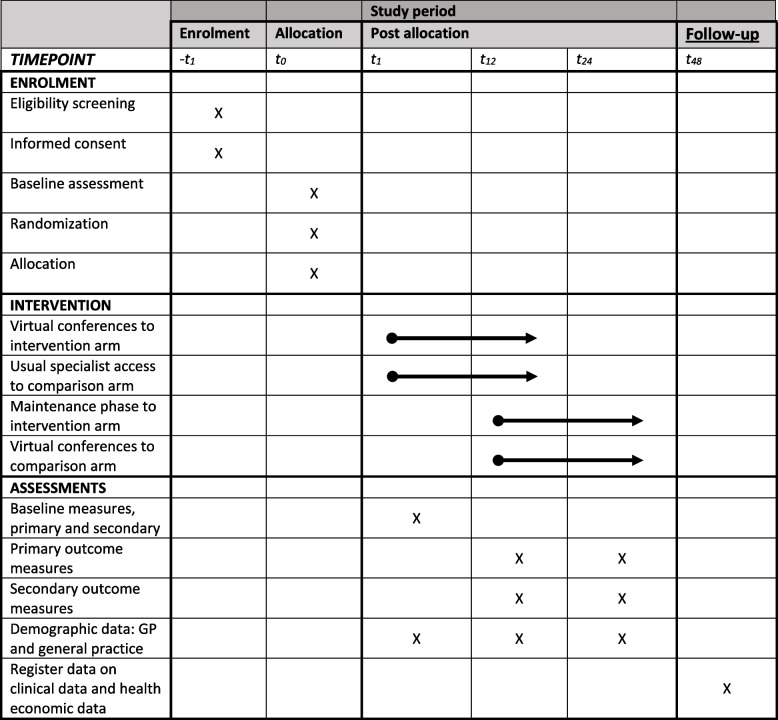
Study schedule

#### Clinical outcomes

Clinical outcomes will be collected from an IT-platform (DEN: “Diabetes Overblik”. ENG: “Diabetes Overview”) used by Danish general practices, which contains anonymous and aggregated diabetes data about patients (Additional file 3 shows screenshots). As per the agreement to participate in the study, each general practice assigns a contact person who will e-mail the screenshot of the data to the project group. Each general practice is renumerated for performing this activity. Data will also be obtained from nationwide Danish registries: The Clinical Laboratory Information System Research Database (LABKA), The Danish National Prescription Registry (DNPR), The Danish National Health Service Register (NHSR), The Danish Civil Registration System (CRS), and The Danish National Patient Register (NPR). Register data will be linked to the unique provider number of each general practice.

#### Competence and organization outcomes

Diabetes competence and organization outcomes in general practice will be collected using an electronic survey (Additional file 4) that was developed with inspiration from a survey similarly concerned with diabetes competence and confidence [37]. The main part of the survey asks respondents about their degree of confidence in managing T2D along seven themes: T2D care in general, T2D care and skills in general, T2D care and cardiovascular disease, T2D care and heart failure, T2D care and blood pressure, T2D care and kidney disease, and T2D care and cholesterol. Other questions concern the respondent, the general practice, organization of diabetes care in the general practice, respondents’ assessment of the virtual specialist conferences via Technology Acceptance Model [38, 39], and collaboration using relational coordination [40]. Answers are given on 1–5 Likert scale. The questionnaire is pilot tested for face validity by GPs.

### Sample and power calculation

The pragmatic nature of the RCT means that the sample size is determined by two contextual circumstances: a formal agreement with PLO-Aarhus and PLO-M to run the study in Aarhus Municipality (cf. above) where the total population of general practices was 100 and the fact that Danish general practices operate as private firms and thus decide themselves if they want to participate in a project. Based on these circumstances along with the study team’s research experiences with recruiting general practices, we used opinion-seeking to derive at the number of 30 general practices that were expected to participate.

We calculated the power for finding a significant difference between the intervention and control group on the three primary clinical outcomes using data from Danish registries and opinion-seeking within the study team. Additional file 5 shows the key parameters and the STATA command we used to calculate the following three power values:

- Power of 0.8870 for Primary clinical outcome 1: Percentage of patients with T2D and ischemic heart disease and/or stroke being treated with GLP1-RA and SGLT2 inhibitor
- Power of 0.9941 for Primary clinical outcome 2: Percentage of patients with micro/macro-albuminuria being treated with ACE or AT2
- Power of 0.9848 for Primary clinical outcome 3: Percentage of patients with LDL > 2.5 mmol/L being treated with Statins.

### Statistical analysis plan

Statistical significance is expressed in 95% two-sided confidence intervals. A *p*-value of < 0.05 will indicate statistical significance.

#### Difference between intervention and control group

The main analysis concerns the difference between the intervention and control group at month 12. Data from the Diabetes Overview and general practice survey are analysed using simple, multiple, and logistic regression analyses. Analysis is done according to the intention-to-treat principle to test if the groups differ in terms of outcome measures. The analysis of survey data will take into account cluster effects using the intra-cluster correlation coefficient. Using *t*-test and *Χ*^2^ tests, depending on the outcome measure, it will be determined if randomization has resulted in systematically different groups. Survey data are tested to determine if questions or indices of questions are internally consistent (Cronbach alpha). In the regression analyses, control variables at the individual (e.g., seniority) and practice level (e.g., form and size of practice) are tested to determine its influence on survey outcomes.

#### Outcomes, process measures, and changes in the individual arms

The two groups (intervention and comparison arms) are analysed on their own using data from the process measures and organizational changes during the study period. The starting point of the intervention group is compared with the outcome measures when the intervention ends (months 0 to 12) and again when the maintenance phase ends (months 12 to 24) to examine the maintenance effect. The intervention period of the control group (months 12 to 24) is analysed to determine the intervention effect. The analyses of the two individual arms are used to study internal changes (before vs. after) and to study if the process measures can explain why the two groups achieve the results they do.

#### Missing data

By applying the above strategies of data collection (e.g., remuneration for providing data, sending reminders) and retention (e.g., general practices signing an agreement to participate), we expect to limit the risk of missing outcome data to a minimum. In case general practices leave the study, we will record the reason(s). To determine if it is relevant to deal with missing data by using multiple imputation (e.g., single value regression analysis), we will follow the recommendation by Jakobsen et al. [41] that the decision relies on being able to answer no to all of the following five key questions, i.e.;

- Is it valid to ignore missing data (rule of thumb: below 5% missing)?
- Too large proportions of missing data (rule of thumb: > 40%)?
- Is data only missing on the dependent variable?
- Is the missing completely at random assumption plausible?
- Is the missing not at random assumption plausible?

In case an answer is yes, we will use observed data only and then thoroughly discuss and report the extent of the missing data and the limitations.

### Process evaluation

Applying a during-trial design nested in the RCT, we will collect process data at the onset, during and after the intervention. The process evaluation will be guided by the MRC framework for conduction and reporting process evaluations [42]. Quantitative process measures are collected by asking endocrinologists to register data in an excel-spreadsheet after each virtual conference. The quantitative process measures will capture two main topics (Table 4): *About the conference*, e.g., information on “number of doctors attended” and “number of patients discussed” and *The outcome of the conference*, e.g., “dialogue about medication changes” or “prevention of inappropriate medication.”

**Table 4:** Process registration

The conference	Number conference (1–4)
	Number of participating general practitioners (number)
	Number of participating practice staff (number)
	Number of patients discussed (number)
Content of the conference	Discussed medical adjustments (yes/no)
	Discussed medical adjustments only according to guidelines (yes/no)
	Prevented inappropriate medication (yes/no)
	Discussed treatment targets (yes/no)
	Number of referred patients to the department of endocrinology? (number)
	Used the “Diabetes Overview”? (yes/no)

To enrich the quantitative analysis, a qualitative process evaluation will study the implementation and use of virtual specialist conferences between general practice and endocrinologists. The analysis will provide knowledge within the domains of intervention adherence, barriers and facilitators for implementing and using virtual specialist conferences, and organizational and contextual influences. The qualitative data are collected at month 12 and 24 using semi-structured interviews with GPs (*n* = 10), practice staff (*n* = 10), and endocrinologists (*n* = 4). The interview guide is based on implementation and change theory [43, 44]. To achieve maximum variation, respondents from general practice are purposefully selected from two types, that is, five GPs and five practice staff from the group of general practices that increased patient and general practice outcomes and five GPs and five practice staff from the group of general practices that achieved negative or unchanged outcomes. The research team performing the interviews and coding the qualitative analysis will not be delivering the intervention. Transcribed interview data is systematically coded using Nvivo (qualitative data processing programme). Coding is first done deductively based on implementation and change theory, and inductively to ensure that all themes in the data are identified. Data are then analysed using the display method to derive learning points and draw conclusions within and across informants and analytical categories [45]. The qualitative data analysis will take place prior to knowing trial outcomes.

### Data management

The study will be performed in accordance with the General Data Protection Regulations (GDPR). The study is reported to The Danish Data Protection Agency (journal no. 1–16-02–398-21), and in accordance with their rules, we report that the expected time for completion of the project and deletion, anonymization, or transfer to the National Archives is 4 October 2026. After data collection, a member of the research team will check data to identify and, where possible, resolve errors prior to analyses being conducted. Two members of the research team will independently prepare data prior to the main analysis. All computers and servers used to manage data and contact with participants will be password protected and housed in secure environments. The Central Denmark Region will provide secure IT systems for secure data management and processing. Register data is managed through IT infrastructure provided by Statistics Denmark. Survey data is collected using SurveyXact. The Central Denmark Region has data management agreements with both Statistics Denmark and SurveyXact. Access to the collected data will only be granted to the research team.

### Monitoring

The project is managed by Steno Diabetes Center Aarhus (SDCA) who will have access to the final dataset. A steering committee consists of the chief executive officer, chief clinician, researchers, TP, AS, and an administrative staff member. On regular meetings, the steering committee will monitor the study procedures and ensure that the trial is being conducted according to the study protocol. The data management team will have clinical, research, and statistical expertise and will consist of the research team, a statistician, and post doc. No interim analyses or auditing are planned since the intervention has been successfully piloted with GPs, and the intervention is not expected to result in any potentially serious outcomes.

## Dissemination plan

Regardless of the magnitude or direction of effect, trial results will be presented at relevant national and international conferences and as published articles in peer-reviewed journals. The analysis is expected to be completed six months after each intervention period ends. The project is expected to result in a minimum of three articles reporting on, respectively, patient outcomes, general practice outcomes, and a qualitative article about the process evaluation. Publication of the study results will be based on the CONSORT extension for pragmatic randomized trials statement [25]. The authorships will follow the International committee of medical journal editors (ICMJE) guidelines. The study results will be disseminated to health care professionals and researchers at (inter)national conferences. The public will learn about the findings in newsletters and via social media. To reach health care policy audiences (e.g. government bodies and unions), plain language findings will be presented at policy maker- and service provider-run conferences.

## Discussion

The intervention relies on randomization of general practices, thereby not targeting a certain type of general practice. The randomization allows for generalizing findings and the qualitative evaluation provides points of learning to GPs across the five Danish regions. The project results also have broader perspectives as the intervention can be applied to other chronic diseases or conditions where dialogue between the hospital and GPs is needed to support the latter in caring for their patients. The results and intervention are also expected to be applicable to other countries with a health system similar to the Danish such as the English NHS. The generalizability is moreover fostered by performing a process evaluation to assess the fidelity of the intervention alongside identification of organizational factors (e.g., how service is delivered) [42].

Given the design of the study, five major limitations apply. First, patient outcomes rely on data retrieved from an IT-platform (Diabetes Overview), and the precision of the outcomes thus depends on the quality of and reporting to the underlying database. Overall, data in Danish registries have high accuracy [46, 47]. To ensure data quality, the first introductory virtual conference serves an important aim and that is getting the GPs to review their list of patients with diabetes (i.e., enter or remove patients) by ensuring that those on the list have the correct diagnostic codes. When patients are registered correctly, data are automatically shown in the Diabetes Overview. The data will be provided by the participating GPs, a task for which they are remunerated, thereby ensuring availability of data.

Second, getting GPs to participate in research projects in Denmark can be difficult because they operate as private for-profit firms, and patient demands means they often work on a tight schedule [30]. Recruitment of GPs during the COVID-19 pandemic is even more challenging [48]. Nevertheless, our recruitment strategy in six steps (e.g., reimbursement and phone calling) are expected to facilitate recruitment. Third, we risk that only GPs participate who are particularly interested in diabetes, thereby resulting in a biased sample. By comparing the sample with the population of GPs and general practices in Aarhus Municipality, we will analyse how similar the sample is to the population.

Fourth, GPs and endocrinologists cannot be blinded to their allocation status. To limit that the behaviour of the control group is influenced, baseline information will not be collected and they are not informed about the data we will collect at month 12. However, participants in the control group can on their own decide to improve the management of T2D by, for example, taking courses and thus move closer to the outcomes of the intervention group. The degree to which this is the case will be captured by asking about changes made during the past year. Fifth, common method bias [49] is considered a small potential risk in the study even though self-reported data (i.e., survey to GPs and practice staff) and administrative health data from the Diabetes Overview (i.e., screen shoots of the primary and secondary outcomes) are collected from the same data source. The risk is moreover considered small because the administrative health data is updated automatically, and the two types of data are collected at different points in time.

## Trial status

This trial was registered on ClinicalTrials.gov with study ID NCT05268081 on 4 March 2022. At the time of submission, recruitment to the trial, which started on 10 December 2021, is ongoing. The anticipated study completion date is September 2024. Protocol version number and date: 1.0, 4 March 2022.

## Supplementary Information

**Additional file 1: Supplementary file 1.** SPIRIT guidelines

**Additional file 2: Supplementary file 2.** Model for the development of the intervention

**Additional file 3: Supplementary file 3.** The “Diabetes Overview” containing aggregated patient data used in general practice

**Additional file 4: Supplementary file 4.** Overview of the questionnaire to general practitioners and practice staff

**Additional file 5: Supplementary file 5.** Key parameters in the power calculations and an example of the STATA command used

**Additional file 6: Supplementary file 6.** Model consent form

**Additional file 7: Supplementary file 7.** CONSORT checklist for pragmatic trials

## Data Availability

Restrictions apply to the availability of these datasets. Register data will be used under license for the current study. Survey data and patient outcomes data are not publicly available as per the written consent signed by general practitioners and endocrinologists. Data are, however, available for research purposes from the authors upon reasonable request and with permission from study participants.

## Abbreviations

T2D: Type 2 diabetes
GP(s): General practitioner(s)
