# Supplementary material for: The effect of virtual specialist conferences between endocrinologists and general practitioners about type 2 diabetes: study protocol for a pragmatic randomized superiority trial
Source: Trials. 2022 Dec 28;23:1059. doi: 10.1186/s13063-022-06961-y (PMC9795951; doi:10.1186/s13063-022-06961-y)

The effect of virtual specialist conferences between endocrinologists and general practitioners about type 2-diabetes: Study protocol for a pragmatic cluster randomized controlled trial

Prætorius, Baymler Lundberg, Søndergaard, Hansen & Sandbæk
Steno Diabetes Center Aarhus

Additional file 2: Model for the development of the intervention

Illustration of the development of the based on a partnership and evidence and theory approach. The logic model is used as a tool to adjust the content of the intervention during the dynamic and iterative process.

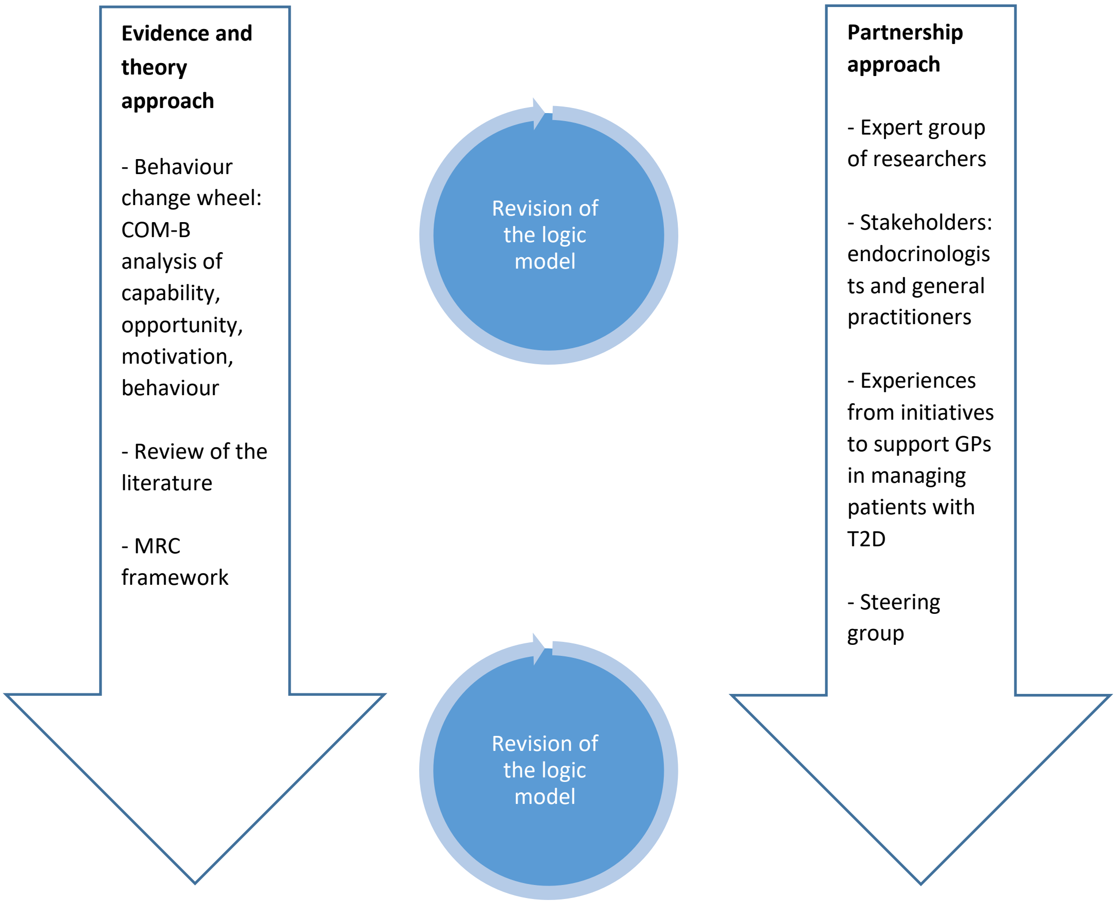

Supplement: Supplementary file 2 — Additional file 2: Supplementary file 2. Model for the development of the intervention [file 13063_2022_6961_MOESM2_ESM.pdf]
