# Supplementary material for: The effect of virtual specialist conferences between endocrinologists and general practitioners about type 2 diabetes: study protocol for a pragmatic randomized superiority trial
Source: Trials. 2022 Dec 28;23:1059. doi: 10.1186/s13063-022-06961-y (PMC9795951; doi:10.1186/s13063-022-06961-y)
Supplement: Supplementary file 3 — Additional file 3: Supplementary file 3. The “Diabetes Overview” containing aggregated patient data used in general practice [file 13063_2022_6961_MOESM3_ESM.pdf]

The effect of virtual specialist conferences between endocrinologists and general practitioners about type 2-diabetes: Study protocol for a pragmatic cluster randomized controlled trial

Prætorius, Baymler Lundberg, Søndergaard, Hansen & Sandbæk
Steno Diabetes Center Aarhus

Additional file 3: Demo screenshot of the "Diabetes Overview" containing aggregated patient data used in general practice [in Danish]

Diabetes prevalence	Diabetes patients	No. patients with yearly check-up	No. patient with diabetes care pathway	No. patients with chronic care code
3.5	7	0 (0%)	4 (57.1%)	1 (14.3%)

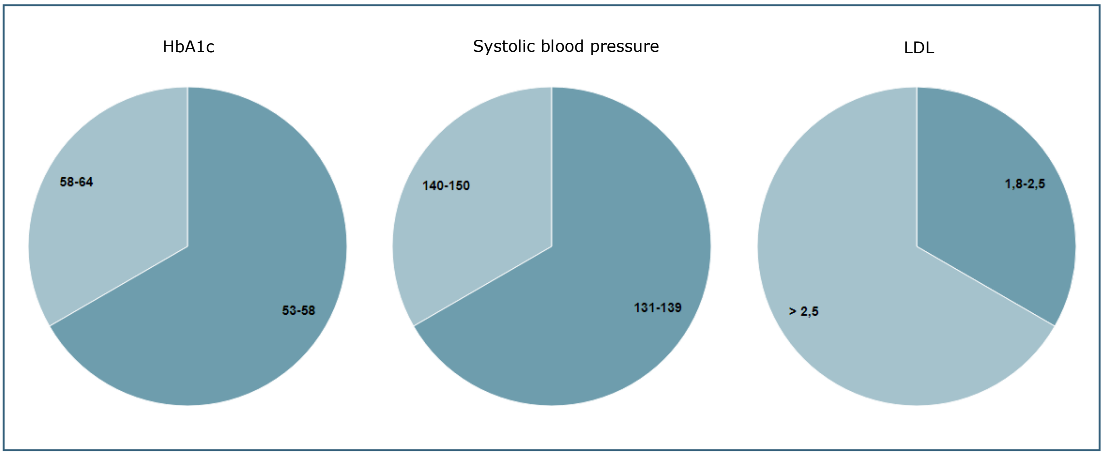

Measure	Name	No.	Share
1 HbA1C	1 Share where HbA1C > 58 mmol/mol	1	14.3%
2 LDL	2 Share where LDL > 2,5 mmol/mol and patient not being treated with metformin	2	28.6%
3 Blood pressure	3 Share where Systolic blood pressure > 140 mmHg	1	14.3%
4 HbA1C continuously elevated	4 Share where HbA1C is continuously elevated	1	14.3%
5 Micro/macro-albuminuria and no ACE/AT2	5 Share where patient has micro or macro-albuminuria and not being treated with ACE/AT2	0	0%
6 eGFR and metformin	6 Share where eGFR<30 ml/min and being treated with metformin	0	0%
7 Ischemic heart disease and GLP1/SGLT2	7 Share with ischemic heart disease and being treated with GLP1/SGLT2	0	0%
