# Supplementary material for: The effect of virtual specialist conferences between endocrinologists and general practitioners about type 2 diabetes: study protocol for a pragmatic randomized superiority trial
Source: Trials. 2022 Dec 28;23:1059. doi: 10.1186/s13063-022-06961-y (PMC9795951; doi:10.1186/s13063-022-06961-y)
Supplement: Supplementary file 4 — Additional file 4: Supplementary file 4. Overview of the questionnaire to general practitioners and practice staff [file 13063_2022_6961_MOESM4_ESM.pdf]

The effect of virtual specialist conferences between endocrinologists and general practitioners about type 2-diabetes: Study protocol for a pragmatic cluster randomized controlled trial

Prætorius, Baymler Lundberg, Søndergaard, Hansen & Sandbæk
Steno Diabetes Center Aarhus

Additional file 4: Overview of the questionnaire to general practitioners and practice staff

Respondent info [4 variables]
Gender; Age; Education; Seniority

Practice info [4 variables]
Type of practice
General practitioners and medical practitioners
Practice staff and diabetes treatment
Patient list size, in general and re. T2DM

Diabetes organization in practice [3 variables]
Management of T2D (3 items)
Organization of work re. T2DM (3/6 item)

Diabetes overview [2 variables]
Use and benefits re. the population of patients with diabetes (3 items)
Use and benefits re. individual patients with diabetes (3 items)

Confidence in managing T2D [7 variables]
The extent to which the GP is confident managing T2D in general (5 items)
The extent to which the GP is skilled in making decisions on T2D in general (7 items)
The extent to which the GP is confident managing T2D and ischemic heart disease, stroke, peripheral artery disease and heart failure (8 items)
The extent to which the GP is confident managing T2D and blood pressure (3 items)
The extent to which the GP is confident managing T2D and kidney disease (7 items)
The extent to which the GP is confident managing T2D and cholesterol (3 items)

Virtual cross-sectorial specialist conferences: technology acceptance model [4 variables, 13 items]
Usefulness; Ease-of-use; Attitude; and Intention to use

Internal and external collaboration about T2D: [8 variables, up to 36 items]
Relational coordination: Shared goals, Shared knowledge; Mutual respect; Frequent communication; Precise communication; Problem-solving communication; timely communication. Use of specialist telephone to the Endocrinology department.

User experiences and changes during the study period [1 variable, 5 items]
