# Supplementary material for: The effect of virtual specialist conferences between endocrinologists and general practitioners about type 2 diabetes: study protocol for a pragmatic randomized superiority trial
Source: Trials. 2022 Dec 28;23:1059. doi: 10.1186/s13063-022-06961-y (PMC9795951; doi:10.1186/s13063-022-06961-y)
Supplement: Supplementary file 5 — Additional file 5: Supplementary file 5. Key parameters in the power calculations and an example of the STATA command used [file 13063_2022_6961_MOESM5_ESM.pdf]

Additional file 5: key parameters in the power calculations and an example of the STATA command used

Additional table: Overview of the key parameters used in the power calculations

		**Parameters used in power calculation**								
**Primary clinical outcome**	**Estimated power at 0.0167 p-value**	**P-value adjusted using Bonferroni correction for multiple primary outcomes**	**General practices in the two study arms**	**Number of patient type in each general practice**	**Assumed percentage at baseline (X_1)**	**Assumed percentage at follow-up (X_1)**	**Intervention effect ($\Delta = X_2 - X_1$)**	**Standard deviation (sd)**	**Additional variance due to clustering using the Intracluster Correlation Coefficient (ICC)**	**Approximative variance of practice specific percentage assumed to be identical**
1: Percentage of patients with T2D and ischemic heart disease and/or stroke being treated with glucagon-like peptide 1 receptor agonists (GLP1-RA) and sodium glucose cotransporter 2 (SGLT2) inhibitor	0.8870	0.0167	$N_{intervention}$ = 15<br>$N_{control}$ = 15	30	30%	42,5%	12,5%	0.09	0.05	36,25%
2: Percentage of patients with micro/macro-albuminuria being treated with Angiotensin-converting-enzyme-inhibitor (ACE) or angiotensin-2-receptorantagonist (AT2)	0.9941	Ibid.	Ibid.	28	75%	90%	15%	0.0737	Ibid.	82,5%
3: Percentage of patients with LDL>2.5 mmol/L being treated with Statins	0.9848	Ibid.	Ibid.	36	40%	55%	15%	0.0854	Ibid.	47,5%

STATA command used to calculate power – exemplified using primary clinical outcome 1:

```
* Number of participating practices
local npractice = 30

* Number of patients per practice
local npat_per_practice = 30

* Approximative variance of practice specific percentage (.3 and .425 have same variance)
local p1 = .3
local p2 = .425

* average percentage
local avprop = (`p1' + `p2') / 2

* Approximative variance of practice specific percentage
local var_bin_in_cluster = `avprop' * (1 - `avprop') / `npat_per_practice'

di `var_bin_in_cluster'

* Assumed value of ICC
local icc = .05

* Based on formula for ICC, we calculate "additional" variance between percentage due to clustering
local var_between = `icc' * `var_bin_in_cluster' / (1 - `icc')

di `var_between'

* Overall SD to be used in power calculation, where practice specific percentage are assumed to follow a normal
distribution with this SD:
local sd_practice_prop = sqrt(`var_between' + `var_bin_in_cluster')

* the power calculation of interest:
power twomeans `p2' `p1', sd(`sd_practice_prop') n(`npractice')
```
