# Supplementary material for: The effect of virtual specialist conferences between endocrinologists and general practitioners about type 2 diabetes: study protocol for a pragmatic randomized superiority trial
Source: Trials. 2022 Dec 28;23:1059. doi: 10.1186/s13063-022-06961-y (PMC9795951; doi:10.1186/s13063-022-06961-y)
Supplement: Supplementary file 6 — Additional file 6: Supplementary file 6. Model consent form [file 13063_2022_6961_MOESM6_ESM.pdf]

Model consent form

Informed consent to participate in a research project on Online specialist conferences about type 2 diabetes

1. Introduction

Thank you for your interest in Online specialist conferences on type 2 diabetes, which is a research project managed by Steno Diabetes Center Aarhus, Aarhus University Hospital.

The aim of the project is to investigate whether a structured process with cross-sectoral virtual specialist conferences between general practitioners and endocrinologists about type 2 diabetes, where they discuss patient treatment and cases, leads to better treatment at patient level, strengthens the collaboration between general practitioners and endocrinologists and increases diabetes competencies in general practice. To understand the use of this type of collaboration in practice, qualitative interviews are conducted with selected general practices.

You have voluntarily given your consent to the processing of your personal data, which is described below in section 2. Appendix 1 summarizes how data is used and your rights in an information form.

You receive this letter so that you can go back at any time and see what you have given your consent to.

2. Consent

By giving your consent, you have given permission for your information to be used for research purposes as described in section 1. Publication of the results of the research project will not contain information that can be attributed to the participants.

The personal information is deleted, anonymised or submitted to the National Archives no later than at the end of the project on 04-10-2026, or earlier if it is no longer necessary and relevant to process the personal information. It is possible that this time will change if the research project is delayed or if data from the project, including your information, is reused in a new research project in accordance with the rules of the Data Protection Act.

Data responsible is Region Midtjylland, Regionshuset Viborg, Skottenborg 26, Postboks 21, 8800 Viborg, CVR number 29190925.

3. Use of consent

By giving your consent, you have given permission for your information to be used as described in section 2 and Appendix 1. The consent has been given voluntarily.

4. Your rights

Consent: You have the right to withdraw a consent.

Correction: You have the right to have incorrect information about you corrected in accordance with the rules of the Data Protection Act.

Deletion: You can request that your information be deleted. If your information is no longer necessary to fulfill the research purpose and deletion of your information does not make it likely to be impossible or seriously prevent the completion of the research project, your information will be deleted.

Restriction of processing: You can request that the processing of your information be restricted. This can e.g. be relevant if your information cannot be deleted, as there may then be a restriction that your information is only stored for a specified period.

Objection: You have the right to object to the processing of your information, unless the processing is necessary to perform a research task in the interest of society.

If you wish to use one or more of your rights, you are welcome to send an email to the project manager: thipra@rm.dk.

You can complain to the Danish Data Protection Agency if you believe that the Data Responsible is disregarding the data protection rules in connection with their use of your information for research purposes / the research project. You are encouraged to contact the project manager and the Data protection officer (dpo@rm.dk) before you complain to the Danish Data Protection Agency, as the case may be resolved or clarified.

5. Certificate of Consent

I have read the foregoing information, or it has been read to me. I have had the opportunity to ask questions about it and any questions that I have asked have been answered to my satisfaction. I consent voluntarily to participate as a participant in this research.

Print Name of Participant__________________

Signature of Participant ___________________

Date: ____

Bilag 1: Informationsskema om brugen af data [in Danish]

Efter databeskyttelsesreglerne har Region Midtjylland pligt til at give dig en række oplysninger i forbindelse med, at dine oplysninger bliver anvendt til forskningsformål. Hvis du har spørgsmål er du velkommen til at kontakte projektleder.	
Dataansvarlig:	Region Midtjylland<br>Regionshuset Viborg, Skottenborg 26, Postboks 21, 8800 Viborg
Projektleder:	Thim Prætorius, thipra@rm.dk
Databeskyttelsesrådgiver:	dpo@rm.dk
Formål:	Projekt anvender et randomiseret design til at undersøge om et struktureret forløb med tværsektorielle og online specialistkonferencer mellem praktiserende læger og endokrinologer om patienter med type 2-diabetes fører til en bedre behandling på patientniveau og et højere niveau af diabeteskompetencer i almen praksis.<br>Dine oplysninger vil udelukkende blive brugt til forskningsformål. Offentliggørelse af forskningsprojektets resultater vil ikke indeholde oplysninger, der kan henføres til de registrerede. Du skal endvidere være opmærksom på, at dine oplysninger kan blive anvendt til andre forskningsprojekter, hvilket i givet fald vil ske i overensstemmelse med databeskyttelseslovens regler.
Grundlaget (hjemlen) i loven:<br><br>Efter databeskyttelsesloven skal enhver behandling af personoplysninger have et lovligt grundlag i loven, og dette grundlag skal oplyses til de personer, hvis oplysninger behandles.	Forskningshjemlen, jf. Databeskyttelseslovens § 10, stk. 2, jf. Databeskyttelsesforordningens art. 6, stk. 1, litra e og art. 89.<br>Du kan læse databeskyttelsesforordningen her: https://www.datatilsynet.dk/generelt-om-databeskyttelse/lovgivning
Eventuelle af modtagere personoplysninger:	Når dine oplysninger behandles til forskningsformål, kan de ikke anvendes til andre formål, og derfor vil eventuelle modtagere af dine personoplysninger altid være forskere.<br>Såfremt dine oplysninger videregives til andre forskningsformål, vil det ske i overensstemmelse med databeskyttelseslovens regler.
Ekstern databehandler:	SurveyXact by Rambøll Management Consulting; Olof Palmes Alle 20; 8200 Aarhus N.<br>Danmarks Statistik Forskermaskiner; Sejrøgade 11; 2100 København Ø
Overførsel til lande uden for EU:	Dine oplysninger vil ikke blive overført til lande uden for EU.
Kategorier af personoplysninger:	Basal sociodemografi (alder, køn, anciennitet) og selvvurderet syn på egne kompetencer i behandlingen af type 2-diabetes. Indsamles på basis af skriftligt samtykke.
Tidsperiode:	Personoplysningerne slettes, anonymiseres eller indsendes til Rigsarkivet senest ved projektets afslutning den 04-10-2026, eller tidligere hvis det ikke længere er nødvendigt og relevant at behandle personoplysningerne.<br>Det er muligt, at dette tidspunkt ændres, såfremt forskningsprojektet forsinkes eller såfremt data fra projektet, herunder dine oplysninger genbruges i et nyt forskningsprojekt i overensstemmelse med databeskyttelseslovens regler.
Rettigheder:	*Berigtigelse*: Du har ret til at få urigtige oplysninger om dig rettet i overensstemmelse med databeskyttelseslovens regler.<br>*Sletning*: Du kan anmode om, at dine oplysninger slettes. Såfremt dine oplysninger ikke længere er nødvendige til at opfylde forskningsformålet, og sletning af dine oplysninger ikke medfører, at det sandsynligvis vil være umuligt eller i alvorlig grad forhindre gennemførelse af forskningsprojektet, vil dine oplysninger blive slettet.<br>*Begrænsning af behandling*: Du kan anmode om, at behandlingen af dine oplysninger begrænses. Dette kan f.eks. være relevant, såfremt dine oplysninger ikke kan slettes, idet at der så kan ske den begrænsning, at dine oplysninger alene opbevares i en nærmere afgrænset periode.<br>*Indsigelse*: Du har ret til at gøre indsigelse mod behandlingen af dine oplysninger, medmindre at behandlingen er nødvendig for at udføre en forskningsmæssig opgave i samfundets interesse.<br>*Samtykke.* Du har ret til at trække et samtykke tilbage.<br>Såfremt du ønsker at bruge en eller flere af dine rettigheder er du velkommen til at sende en mail herom til projektleder.
Klage:	Du kan klage til Datatilsynet, såfremt du mener, Dataansvarlig tilsidesætter databeskyttelsesreglerne i forbindelse med deres behandling af dine oplysninger til forskningsformål/forskningsprojektet. Du opfordres til at kontakte projektleder o/e DPO-rådgiveren, inden du klager til Datatilsynet, da sagen eventuelt vil kunne løses eller afklares.
Projektanmeldelse:	Forskningsprojektet er anmeldt internt i Region Midtjylland (sagsnr. 1-16-02-398-21), som har fastsat nærmere vilkår for projektet til beskyttelse af den registreredes privatliv.<br>De Videnskabsetiske Komitéer for Region Midtjylland (sagsnr. 1-10-72-274-21) oplyser: Spørgeskemaundersøgelser er undtaget fra anmeldelse i medfør af komitéloven § 14, stk. 2, og projektet skal derfor ikke anmeldes til komitéen, jf. samme lov § 14, stk. 1.
